# Markers of Central Neuropathic Pain in Higuchi Fractal Analysis of EEG Signals From People With Spinal Cord Injury

**DOI:** 10.3389/fnins.2021.705652

**Published:** 2021-08-26

**Authors:** Keri Anderson, Cristian Chirion, Matthew Fraser, Mariel Purcell, Sebastian Stein, Aleksandra Vuckovic

**Affiliations:** ^1^Biomedical Engineering Division, University of Glasgow, Glasgow, United Kingdom; ^2^School of Computing Science, University of Glasgow, Glasgow, United Kingdom; ^3^Queen Elizabeth National Spinal Injuries Unit, Queen Elizabeth University Hospital, Glasgow, United Kingdom

**Keywords:** Higuchi fractal dimension, non-oscillatory features, central neuropathic pain, EEG, movement imagination, spinal cord injury

## Abstract

Central neuropathic pain (CNP) negatively impacts the quality of life in a large proportion of people with spinal cord injury (SCI). With no cure at present, it is crucial to improve our understanding of how CNP manifests, to develop diagnostic biomarkers for drug development, and to explore prognostic biomarkers for personalised therapy. Previous work has found early evidence of diagnostic and prognostic markers analysing Electroencephalogram (EEG) oscillatory features. In this paper, we explore whether non-linear non-oscillatory EEG features, specifically Higuchi Fractal Dimension (HFD), can be used as prognostic biomarkers to increase the repertoire of available analyses on the EEG of people with subacute SCI, where having both linear and non-linear features for classifying pain may ultimately lead to higher classification accuracy and an intrinsically transferable classifier. We focus on EEG recorded during imagined movement because of the known relation between the motor cortex over-activity and CNP. Analyses were performed on two existing datasets. The first dataset consists of EEG recordings from able-bodied participants (*N* = 10), participants with chronic SCI and chronic CNP (*N* = 10), and participants with chronic SCI and no CNP (*N* = 10). We tested for statistically significant differences in HFD across all pairs of groups using bootstrapping, and found significant differences between all pairs of groups at multiple electrode locations. The second dataset consists of EEG recordings from participants with subacute SCI and no CNP (*N* = 20). They were followed-up 6 months post recording to test for CNP, at which point (*N* = 10) participants had developed CNP and (*N* = 10) participants had not developed CNP. We tested for statistically significant differences in HFD between these two groups using bootstrapping and, encouragingly, also found significant differences at multiple electrode locations. Transferable machine learning classifiers achieved over 80% accuracy discriminating between groups of participants with chronic SCI based on only a single EEG channel as input. The most significant finding is that future and chronic CNP share common features and as a result, the same classifier can be used for both. This sheds new light on pain chronification by showing that frontal areas, involved in the affective aspects of pain and believed to be influenced by long-standing pain, are affected in a much earlier phase of pain development.

## 1. Introduction

Central neuropathic pain (CNP) is an excruciating secondary consequence of spinal cord injury (SCI). CNP is a chronic condition caused by an injury to the somatosensory system (Jensen et al., 2011) and affects more than 50% of all people with SCI (Siddall et al., 2003; Finnerup, 2013) and subsequently, their quality of life (Mann et al., 2013). There is currently no cure for CNP with primary treatments focussed on pain management rather than elimination. It is thought that the development of CNP in SCI is the result of neuronal hyperexcitability that develops following damage to the spinal cord, eventually resulting in the perception of pain (Wasner et al., 2008; Zeilig et al., 2012; Finnerup et al., 2014). At the supraspinal level, many studies have shown a relationship between CNP and the reorganisation of the sensorimotor cortex because of sensory losses and changes to the central nervous system (CNS) caused by injury (Wrigley et al., 2009; Gustin et al., 2010). While most neuroimaging studies have been based on fMRI, changes in the cortical pain matrix can also be detected using electroencephalography (EEG). For example, at the cortical level, pain is known to cause thalamocortical dysrhythmia, which presents as increased theta and beta band EEG power, reduced alpha band power and slowed-down dominant alpha frequency (Sarnthein et al., 2006; Stern et al., 2006; Boord et al., 2008; Jensen et al., 2013; Vuckovic et al., 2014, 2018a).

With regards to SCI related pain, fMRI studies on people who imagined movement of otherwise paralysed limbs, showed that the sensory-motor cortex can be over-active in people with neuropathic pain (Gustin et al., 2010). Based on EEG analysis, in a similar experimental paradigm based on movement imagination, our group defined dynamic markers of chronic SCI related to CNP, that changed with time and were frequency-specific (Vuckovic et al., 2014). In a subsequent study, on people with recent (subacute) SCI, applying a similar experimental paradigm, we discovered EEG prognostic markers of “future” CNP, in participants with SCI who developed pain within 6 months following EEG recording (Jarjees, 2017). A resting state EEG of these participants also contained highly discriminable oscillatory markers of future pain in the theta, alpha and beta bands (Vuckovic et al., 2018a) based on which we developed transferable classifiers (Vuckovic et al., 2018b). These classifiers were able to predict, with an average accuracy of 86%, the risk of developing pain for participants outside of the training set.

Alterations in human EEG activity following spinal injury can be analysed using sophisticated linear and non-linear methods to assess for changes to brain function. Linear methods based on time-frequency analysis are best suited to describe the oscillatory, frequency-specific nature of EEG signals. However more recently non-linear methods, originating from the chaos theory, have shown to provide a separate and partly independent set of information about the signals (Bhattacharya, 2000; Natarajan et al., 2004; Acharya et al., 2005; Stam, 2005). Of these non-linear methods, one most frequently used to analyse and define non-oscillatory EEG signals is fractal dimension (Accardo et al., 1997), which acts as a measure of signal complexity (Higuchi, 1988; Katz, 1988; Petrosian, 1995; Kalauzi et al., 2005). Higuchi's fractal dimension (HFD) (Higuchi, 1988) has been applied extensively to EEG signals in various studies which characterise EEG in healthy participants and with a range of neurological problems such as Alzheimer's, brain injury, dementia, epilepsy, and stroke (Henderson et al., 2000; Li et al., 2005; Spasic et al., 2011; Smits et al., 2016). Fractal values have repeatedly shown to be lower in people suffering from brain disorders than healthy people (Staudinger and Polikar, 2011; Ahmadlou et al., 2012; Kesić and Spasić, 2016), where a loss of complexity in brain activity leads to the neural system of the brain becoming less flexible and efficient in processing (Goldberger et al., 2002; Zappasodi et al., 2014).

HFD has previously been used to classify pain evoked potentials in the EEG of healthy adults (Tripanpitak et al., 2020) in which evidence was presented that using non-linear features, such as HFD, could classify between different perception levels from non-invasive electrical stimulation. On the basis that oscillatory features of CNP in SCI have been established we consider that, using HFD as a measure for signal analysis, we may be able to find classifiable, non-oscillatory markers of CNP using fractals. Complimentary to this, features from entropy during resting state EEG, a non-linear analysis technique, have been used to classify between different phases of migraine that precede/follow pain (Cao et al., 2018). This indicates the possibility for classifiable non-linear EEG features.

Consequently, we aimed to look for non-linear EEG features of pain during movement imagination (MI), given that FD (Fractal Dimension) markers of movement-related tasks have previously been determined in the EEG of healthy participants. Phothisonothai and Nakagawa (2007) were able to extract fractal information specific to four different motor imagery tasks presented to healthy participants. Their participants showed that the FD values of proposed motor imagery tasks existed using different fractal algorithms, for which they offer benefits in different applications. They indicated that the Higuchi method is suited for evaluating the FD of EEG data with high precision.

Results of these studies indicate that it might be possible to apply HFD analysis to movement-related EEG to diagnose CNP in a similar way that the oscillatory activity of the brain is able to do (Sarnthein et al., 2006; Stern et al., 2006; Boord et al., 2008; Jensen et al., 2013; Vuckovic et al., 2014, 2018a). The potential advantage of HFD-based, non-linear features being to increase the repertoire of available analyses on the EEG of people with subacute SCI, where having both linear and non-linear features for classifying pain may ultimately lead to higher classification accuracy and an intrinsically transferable classifier. In the current study, we explore the application of HFD to identify classifiable, movement-related EEG features.

Having considered the results of the discussed studies, we establish two novel aspects to this study: (i) identifying non-linear features of CNP (ii) using non-linear features based on induced dynamic activity, rather than resting state, to classify a neurological disorder, such as pain. Our previous research showed that chronic and subacute CNP have some common markers, as described by the oscillatory EEG activity, and that these markers are frequency specific. We hypothesise therefore that if common markers exist, a diagnostic classifier developed on EEG of people with chronic pain could also be applied as a prognostic pain marker in a subacute state. Furthermore, because optimal frequency bands for each participant do not need to be identified, unlike the case with dynamic EEG responses, we also hypothesise that a classifier based on non-oscillatory features may be less sensitive to any differences between diagnostic and prognostic markers than oscillatory features. We test therefore the aforementioned diagnostic classifier on EEG data of people with subacute SCI in order to distinguish those who are about to develop pain from those who are not going to develop pain.

## 2. Materials and Methods

### 2.1. Datasets

Two pre-existing datasets of cue-based MI recordings were used for this study (Vuckovic et al., 2014, 2018b).

Dataset 1 (Vuckovic et al., 2014) was recorded with a 61-channel EEG (Synamp 2; NeuroScan, Charlotte, NC) at 250 Hz and involves three groups of participants: 10 able-bodied volunteers (3 F, 7 M, age 39.1 ± 10.1), referred to as group **AB**, 10 chronic paraplegic participants with diagnosed CNP below the level of their SCI (3 female [F], 7 male [M], age 45.2 ± 9.1 [mean ± standard deviation], 5 incomplete SCI), referred to as chronic SCI participants with pain (**cPWP**), 10 participants with chronic SCI and no pain (2 F, 8 M, age 44.4 ± 8.1, 3 incomplete SCI), referred to as chronic SCI participants with no pain (**cPNP**). All participants with SCI were at least 1 year post-injury, with a spinal lesion at or below T1. Inclusion criteria for participants with CNP were a positive diagnosis of CNP and a treatment history of CNP for at least 6 months. All participants with CNP reported their pain to be in their lower abdomen and legs.

Dataset 2 (Vuckovic et al., 2018b) was recorded with a 48-channel EEG at 256 Hz and involves subacute (less than 6 months post-injury) paraplegic and tetraplegic participants who showed no CNP symptoms at the time of the study. Participants were assessed for CNP 6 months after recording and assigned to one of two groups based on their diagnosis. 10 participants with SCI developed CNP (4 tetraplegic, 4 incomplete SCI); they will collectively be referred to as subacute SCI participants that developed CNP (**sPDP**). 10 participants with SCI did not develop pain (4 tetraplegic, 6 incomplete SCI); they will collectively be referred to as subacute SCI participants with no pain (**sPNP**).

**Figure 1:**
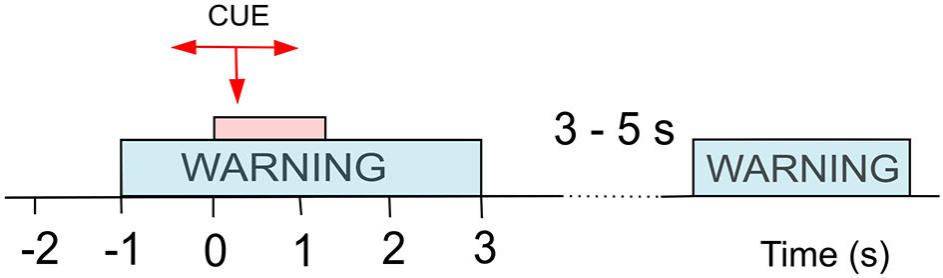
Experimental setup for cue-based motor imagery (MI) tasks as used in both Dataset 1 (Vuckovic et al., 2014) and Dataset 2 (Vuckovic et al., 2018b). At *t* = −1 s a warning signal (a cross) appeared on a computer screen, followed by a cue (an arrow) at *t* = 0 s. The cue stayed on the screen until *t* = 1.25 s while the warning stayed until *t* = 3 s. A volunteer was asked to perform repetitive imagination of movement from *t* = 0 s until the warning disappeared at *t* = 3 s. Different arrows indicated motor imagery of different limbs.

**Figure 2:**
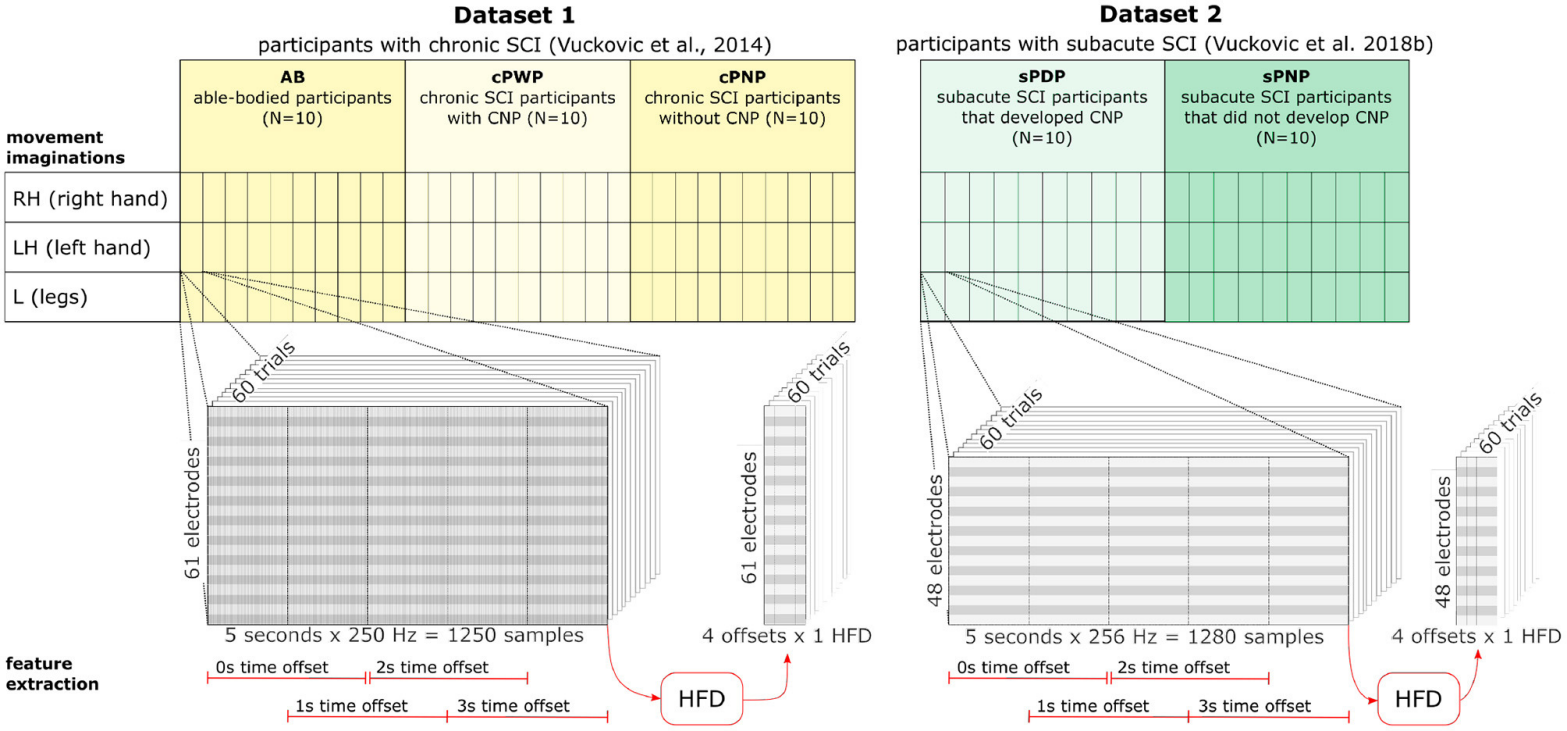
Dataset organisation **(top)** and feature extraction **(bottom)**. Dataset 1 includes data from three groups (AB, cPWP, cPNP) with 10 participants in each group. Each participant's MI of three different movements (RH, LH, L) were recorded with 60 repetitions per movement conducted in semi-random order. Each recording contains 5 s data sampled at 250 Hz at 61 electrode positions. Dataset 2 includes data from two groups (sPDP, sPNP) with 10 participants in each group. Each participant followed the same experimental protocol as in Dataset 1. Each recording contains 5 s data sampled at 256 Hz at 48 electrode positions. See section 2.1 for more detail. HFD features were extracted at each electrode position from four temporal windows of 2 s length, each offset by 50%. See section 2.2 for more detail.

Both datasets were recorded following the same experimental design (Figure 1), and recorded data was pre-processed in the same manner. Participants were presented with a visual warning (a cross) at *t* = −1 s, followed by an initiation cue (an arrow) after 1 s (at *t* = 0 s) which remained visible for 1.25 s. Participants were instructed to imagine movement of the left hand (←), right hand (→) or both feet (↓) until the disappearance of the warning cue (at *t* = 3 s). A total of 60 repetitions were recorded from each participant for each limb movement in a semi-random order. An illustration of the total available data and its organisation is given in Figure 2 (top). Whilst most people with paraplegia and CNP experience pain in their legs, i.e., below the level of their injury, it is of interest to examine data from potentially non-painful limbs, i.e., MI of limbs above the level of injury. Dynamic EEG changes during pain have previously been observed in the sensory-motor cortex (Gustin et al., 2010), which is activated during MI. Thus, we include data recorded during imagined hand movements in individuals with paraplegia to explore whether discriminatory non-linear information exists in motor-cortex activation. For details on the experimental paradigm and on data pre-processing please refer to Vuckovic et al. (2014).

In both studies, informed consent was obtained from all participants, and ethical approval was obtained from the National Health Service ethical committee for the patient groups and from the university ethical committee for the able-bodied group.

### 2.2. Feature Extraction

As features for data analysis and classification, we explore Higuchi's fractal dimension (HFD) of data from individual EEG channels (Higuchi, 1988). The fractal dimension *D* is a statistical measure relating signal complexity to the scale at which a signal is measured, with higher values of *D* corresponding to higher signal complexity. For timeseries, data *D* ∈ [1, 2]. As HFD analysis is more effective and more efficient on shorter time windows (Kesić and Spasić, 2016) we use EEG data cropped to 2.0 s windows with 50% overlap. We consider time windows with 0, 1, 2, and 3 s offset from the start of the recording, respectively, to investigate whether HFD at specific stages of cue-based MI can offer more discriminatory information about participants with SCI and CNP. This could identify whether there exist smaller time intervals of the recordings that could help train a better classifier on HFD features compared to classification using the full time series of each repetition.

The HFD is estimated as follows. First, the length *L*(*k*) of a timeseries *S*(*t*), *t* = 1, …, *N* is calculated using Equations (1)–(2) for exponentially increasing values of *k* = 2, …, ≤ *k*_*max*_.

(1) $$\begin{array}{l} L_{m}\left(k\right)=\left(\overset{\left\lfloor \frac{N-m}{k}\right\rfloor }{\underset{i=1}{\sum }}|S\left(m+ik\right)-S\left(m+\left(i-1\right)k\right)|\right)\frac{1}{k}\frac{N-1}{\left\lfloor \frac{N-m}{k}\right\rfloor } \end{array}$$

(2) $$\begin{array}{l} L\left(k\right)=\frac{1}{k}\overset{k-1}{\underset{m=0}{\sum }}L_{m}\left(k\right) \end{array}$$

Then, the fractal dimension *D* is estimated from the slope of the linear least squares fit of *L*(*k*) onto *k* on a doubly logarithmic scale such that, for statistically self-similar curves, *L*(*k*) ∝ *k*^−*D*^. We used an open source Python implementation^1^ and set *k*_*max*_ = 7. Our choice of *k*_*max*_ = 7 was motivated by previous work showing particularly accurate estimation of the fractal dimension in EEG signals with *k*_*max*_ near 6 (Accardo et al., 1997). We extract HFD features from each temporal window, at each electrode position, in each repetition of MI recorded from all participants (see Figure 2, bottom).

**Figure 3:**
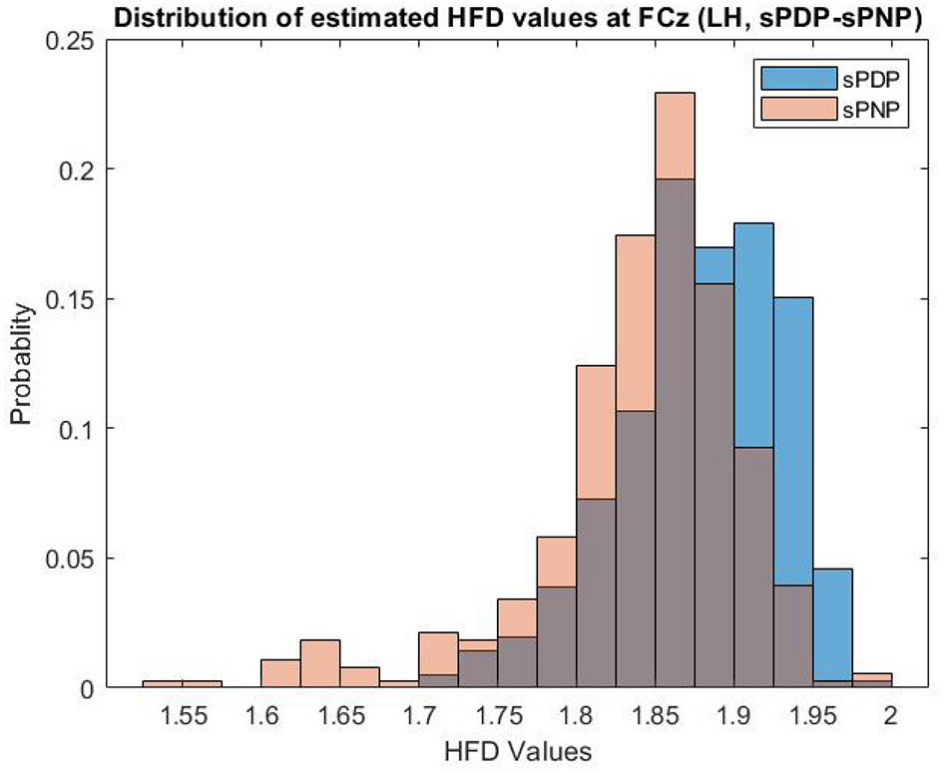
Example distributions of HFD estimated from EEG data of both participant groups in Dataset 2 at one electrode (FCz) during Left Hand (LH) movement imaginations. HFD values range from 1.5 to 2.0 and are most frequently close to 1.85, with a larger proportion of HFD features extracted from EEG data of participants in the group sPNP at lower values, and a larger proportion of HFD features extraced from EEG data of participants in the group sPDP at higher values.

Example distributions of HFD estimated from EEG data of both participant groups in Dataset 2 at one electrode (FCz) during Left Hand (LH) movement imaginations are shown in Figure 3. HFD values range from 1.5 to 2.0 and are most frequently close to 1.85, with a larger proportion of HFD features extracted from EEG data of participants in the group sPNP at lower values, and a larger proportion of HFD features extraced from EEG data of participants in the group sPDP at higher values.

### 2.3. Analysis

**Figure 4:**
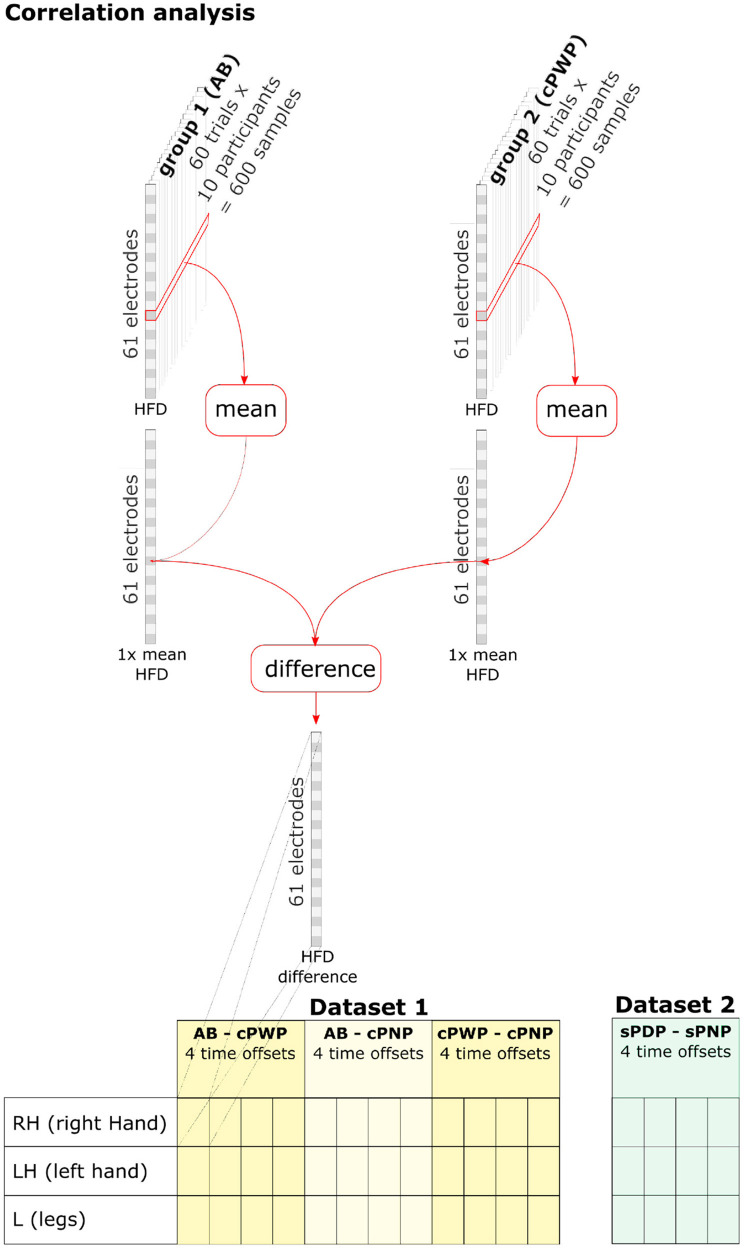
HFD features were aggregated across all trials and participants within each group by estimating their mean value. Group-level differences in HFD features were computed separately for each pair of groups (within each dataset), each time offset, each MI, and each electrode position. See section 2.3 for details.

To analyse spatio-temporal variations in HFD within participant groups, HFD features were aggregated across all trials and participants within each group by estimating their mean value (see Figure 4, top). A separate group mean was estimated for each group, each time offset, each MI, and each electrode position, respectively. Group-level differences in mean HFD features were computed separately for each pair of groups (only considering pairings within each dataset), each time offset, each MI, and each electrode position. Figure 4 (bottom) illustrates a full enumeration of conditions that were explored.

**Figure 5:**
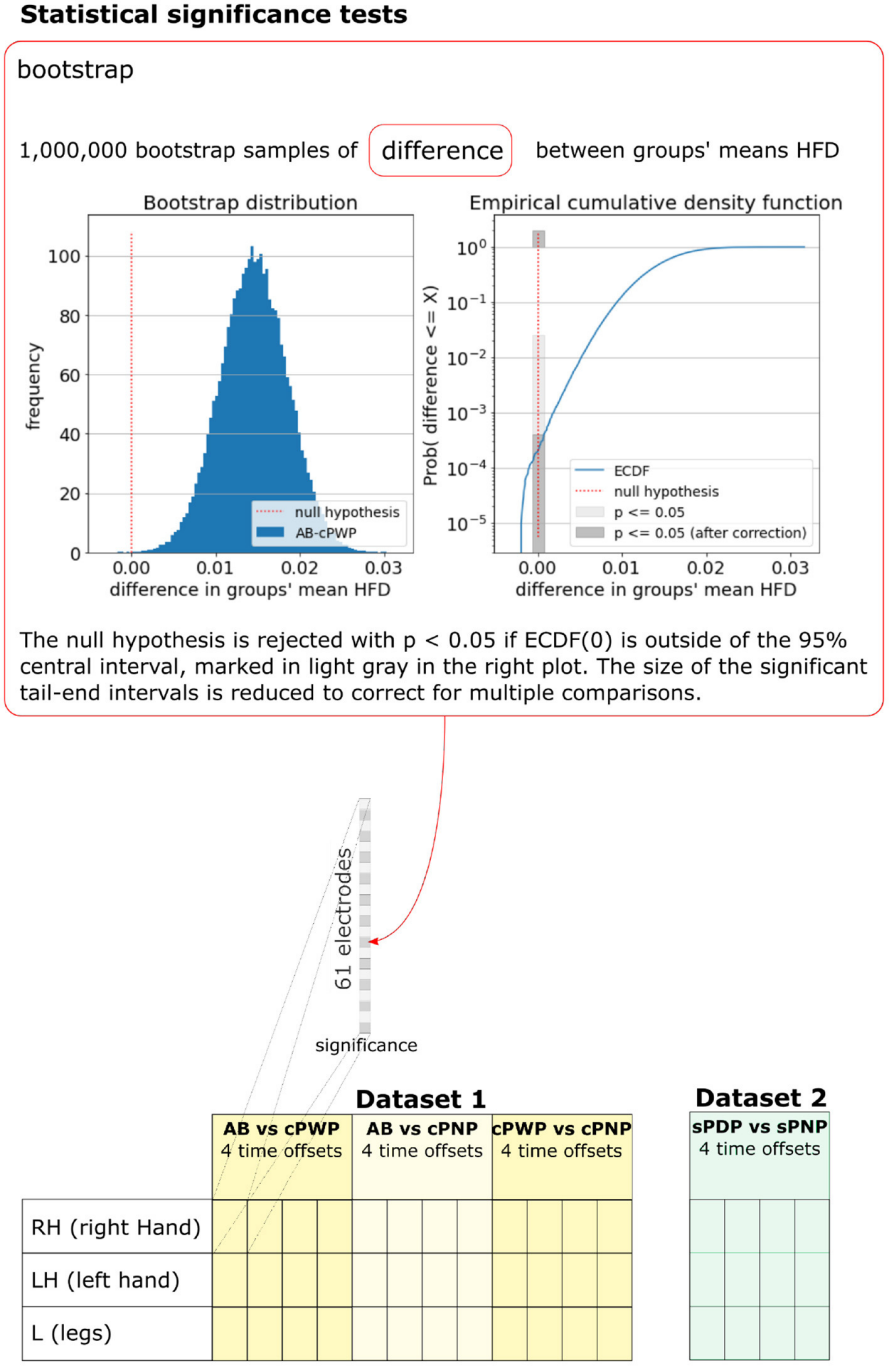
Bootstrap was used to test for statistical significance of differences in group-mean HFD features. The bootstrap sample distribution of differences between groups, the corresponding empirical cumulative density function, and significance thresholds are illustrated for one example pair of groups (AB vs. cPWP), time offset (3 s), MI (RH), and electrode position. A full enumeration of all pairwise differences that were tested is illustrated at the bottom. See section 2.3 for details.

As HFD features were not normally distributed, the statistical significance of differences in the mean HFD features between groups was tested using the bootstrap method. *M* = 1, 000, 000 samples of differences in mean HFD features were drawn by first generating *M* synthetic datasets of HFD features, where (60 trials × 10 participants = 600) HFD features were sampled with replacement from each group, and then estimating their group mean as above. The empirical cumulative density function of *M* samples $\text{ECDF}\left(\tau \right)=\frac{\#\left\{x\leq \tau \right\}}{M}$ approximates the cumulative density function Prob(*x* ≤ τ). The null hypothesis *H*_0_:μ_*a*_ − μ_*b*_ = 0 in the two-tailed test is not rejected with probability *p* if $\frac{p}{2}<\text{ECDF}\left(0\right)<1-\frac{p}{2}$, and rejected with probability *p* otherwise. We tested for *p* = 0.05. To account for spurious results due to multiple comparisons, one for each EEG channel, the significance threshold was adjusted using Bonferroni Correction by multiplying *p* with $\alpha =\frac{1}{61}$ for Dataset 1 and with $\alpha =\frac{1}{48}$ for Dataset 2, respectively. This process is illustrated in Figure 5, which also enumerates all pairwise comparisons we considered.

### 2.4. Classification

In addition to analysing correlations between HFD of individual EEG channels and participant groups we explore the potential of HFD as features for classification using linear Support Vector Machines (SVM). SVMs have been shown to outperform other methods in a large number of EEG classification problems, which is attributed, in part, to SVMs' ability to classify relatively small datasets (Lotte et al., 2007). SVM classifier training involves representing the training data points in a multi-dimensional vector space and finding a hyperplane that separates the data points belonging to different classes, while maximising the distance between the hyperplane and the data points closest to it, which are referred to as 'support vectors' in Schölkopf et al. (2000). Here, we used the Sklearn implementation^2^ of the ν-SVC classifier by Schölkopf et al. (2000).

**Figure 6:**
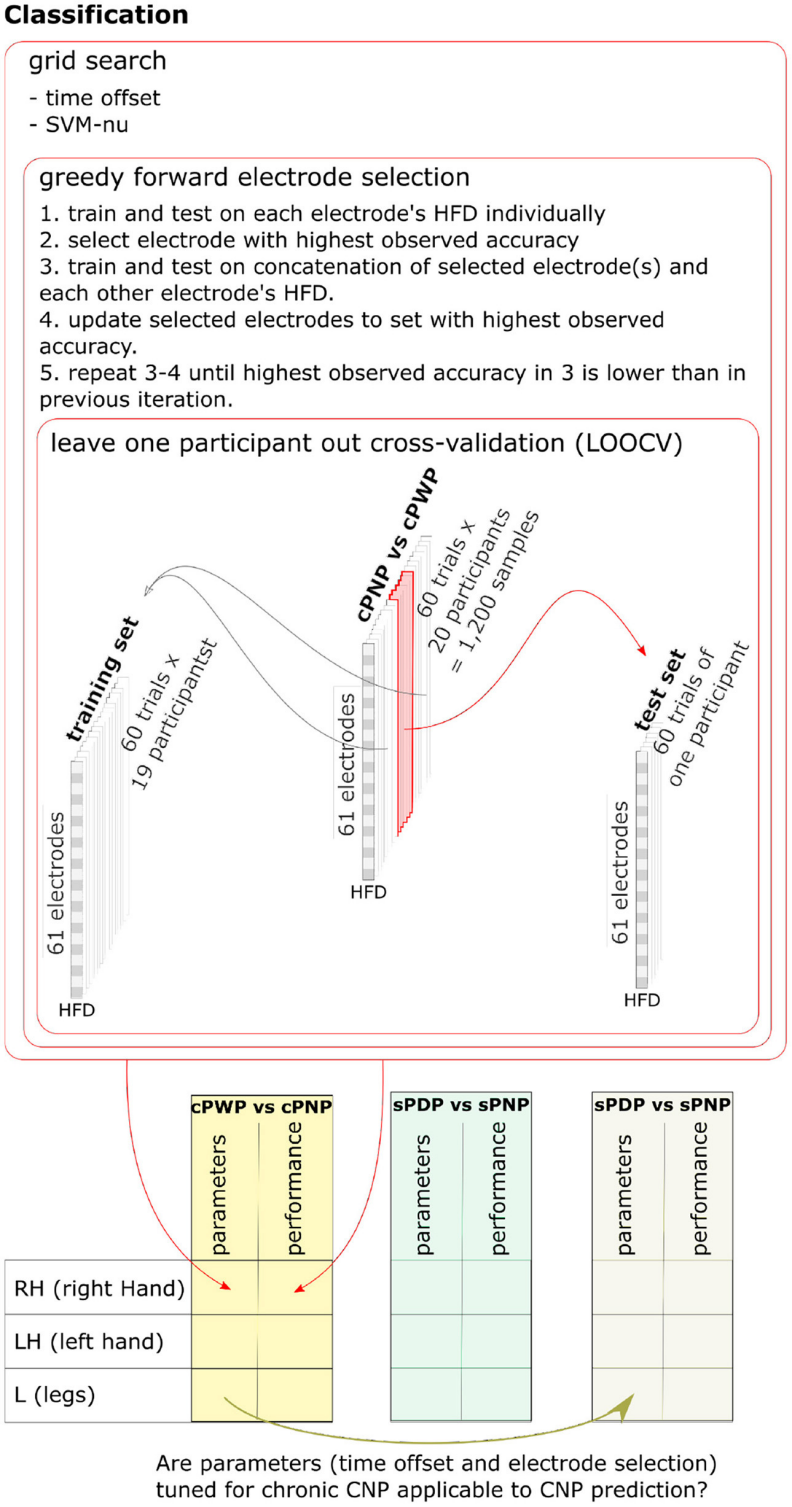
Nested training procedure **(top)** and enumeration of all pair-wise classifiers **(bottom)**. Nested leave-one-subject-out-cross-validation is used to estimate classifier generalisation performance. Hyper-parameters are optimised using grid search and cross-validation on the training set, with greedy forward selection of electrodes used as input to the classifier. One pairwise classifier for each MI task was trained on Dataset 1 (cPWP vs. cPNP) and on Dataset 2 (sPDP vs. sPNP). We also explored whether additional knowledge gained from diagnostic classifier training can be transferred to the prognostic classifier, by using channels and time-window offsets that performed best on cPWP vs. cPNP to optimise classifiers on sPDP vs. sPNP. See section 2.4 for details.

The SVM input vector space was constructed from HFD features extracted from a subset of EEG channels. Hyper-parameter search thus involved optimising this subset of EEG channels, optimising the ν-SVC hyper-parameter ν ∈ (0, 1], which bounds the fraction of support vectors and margin errors, and optimising the choice of time offset from the start of EEG recording. We optimised these jointly via nested cross-validation on the training set, using a wrapper method for EEG channel selection known as *greedy forward feature selection* (Deng and Moore, 1998; Figueroa and Neumann, 2014). Greedy forward feature selection starts by estimating the cross-validation accuracy of a ν-SVC trained on HFD features from each EEG channel independently. The algorithm adds the channel with highest observed mean accuracy to the initially empty *selected* set. In each subsequent iteration, cross-validation accuracy of a ν-SVC trained on each non-selected channel in conjunction with all the features in the *selected* set is evaluated, and the best performing further channel is added to the *selected* set. The procedure halts when there is no further channel to add without reducing classification accuracy. For a more detailed description and a pseudo-algorithm see Chapter 7.3 in Deng and Moore (1998). The parameters ν and the time offset are determined via grid-search and cross-validation on the training set, where the subset of channels used is re-optimised for each parameter value. Figure 6 illustrates the nested training procedure (top) and enumerates all pair-wise classifiers we consider (bottom).

To explore whether HFD features can be used as diagnostic markers for CNP, we trained SVM classifiers on Dataset 1 to discriminate between cPWP and cPNP. To test transferability, classifiers were trained on examples from all but one participant and evaluated on the held-out participant repeatedly with each participant being held out once. To explore whether HFD features can be used as prognostic markers for CNP in participants with subacute SCI, we trained classifiers on Dataset 2 to discriminate between sPDP and sPNP. We also explored whether additional knowledge gained from diagnostic classifier training can be transferred to the prognostic classifier, by using channels and time-window offsets that performed best on Dataset 1, cPWP vs.cPNP classification to optimise classifiers on Dataset 2, sPDP vs. sPNP. Using a multiway analysis of variance (ANOVA) we tested for statistically significant main effects of the time window offset and the type of movement imagination on classification accuracy, as well as their interaction effect.

In addition to evaluating mean accuracy per trial, we explored the potential for using repeated measurements to improve prediction performance by aggregating predictions for each participant via majority voting: determining a participant-level class prediction based on the class that was most frequently predicted across all individual recordings.

## 3. Results

### 3.1. Correlation of HFD With Participant Groups

**Figure 7:**
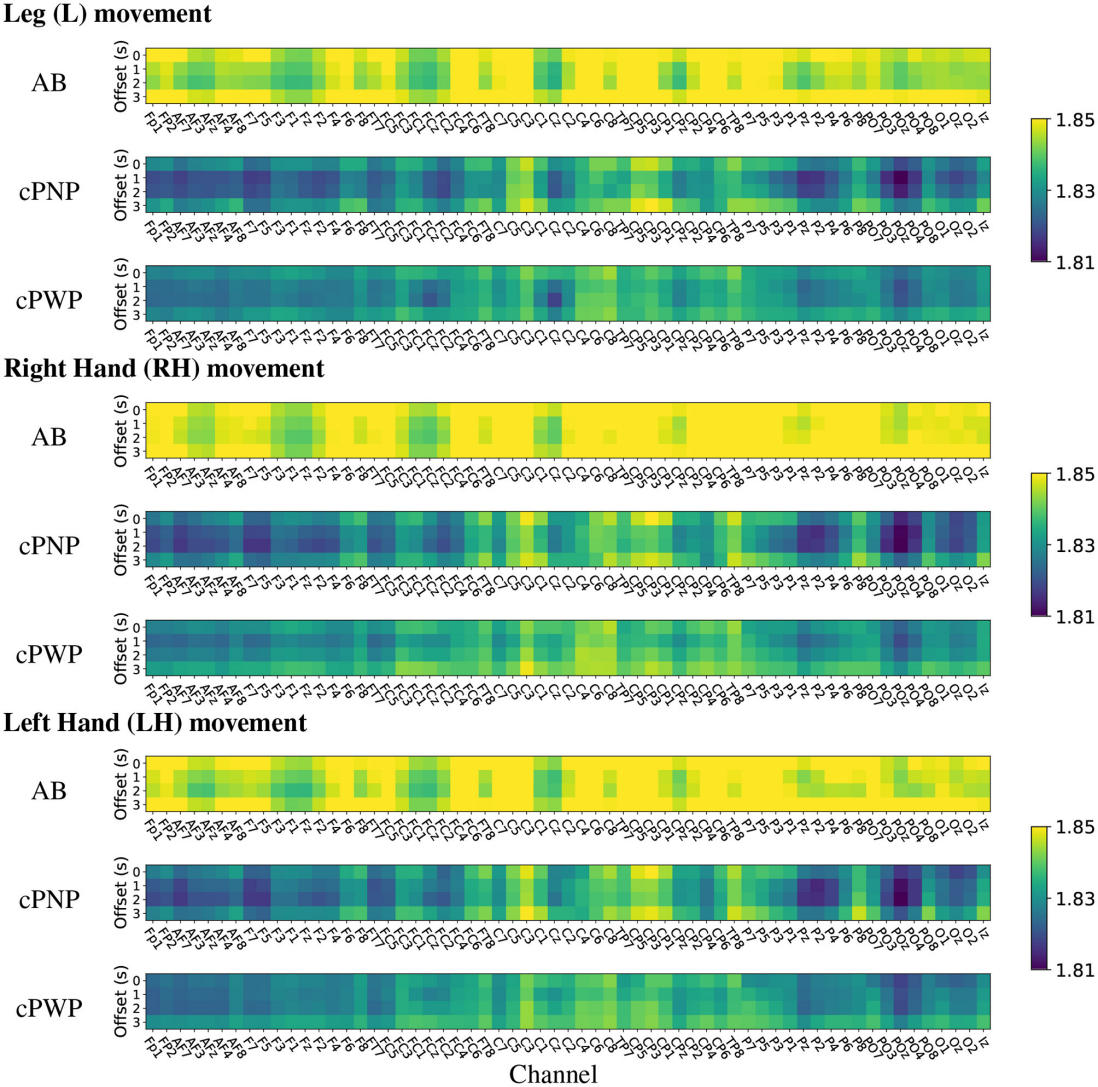
(Dataset 1) Heat maps of group-wise mean Higuchi Fractal Dimension features extracted from EEG signals recorded during movement imagination from able-bodied volunteers (AB), participants with chronic SCI and no pain (cPNP), and participants with chronic SCI and chronic CNP (cPWP). Colours represent low (blue), medium (green), and high (yellow) mean HFD values ordered by channel along the x-axis and by time offset along the y-axis.

Heat maps of group-wise mean HFD features estimated on Dataset 1 are shown in Figure 7. The heat maps provide a visual representation in 2 dimensions of the group-wise mean complexity of the signal at each channel location for each time offset. The start of each *time window* is indicated on the y-axis of the heat maps, with 0 s offset coinciding with the onset of the warning signal. Each column represents the activity at each of the 61 EEG channels recorded in Dataset 1, for all repetitions of either Right Hand (RH), Left Hand (LH) or Leg (L) movement imaginations (MI) from participants in groups; able-bodied volunters (AB), participants with chronic SCI and chronic CNP (cPWP) and participants with chronic SCI and no pain (cPNP), respectively. Bright yellow colours indicate higher group-wise mean HFD corresponding to greater signal complexity whilst blue colours represent lower complexity.

Our group-wise mean HFD analyses demonstrate that AB have, on average, higher mean HFD than both participant groups with SCI (cPWP and cPNP). The heat maps of mean HFD are similar for MI of all three limbs with highest values of mean HFD over the left motor cortex. Mean HFD from both participant groups with SCI are similar for MI of each limb with highest mean HFD for the left (dominant) sensory-motor cortex at locations C1, C3, Cp1, Cp3. There is no systematic difference in mean HFD across time windows which start at different times with respect to the cue-based movements. The largest difference between participant groups with SCI was observed over the parietal area (secondary sensory cortex) which is part of the pain matrix (also see bottom row of **Figure 9**).

**Figure 8:**
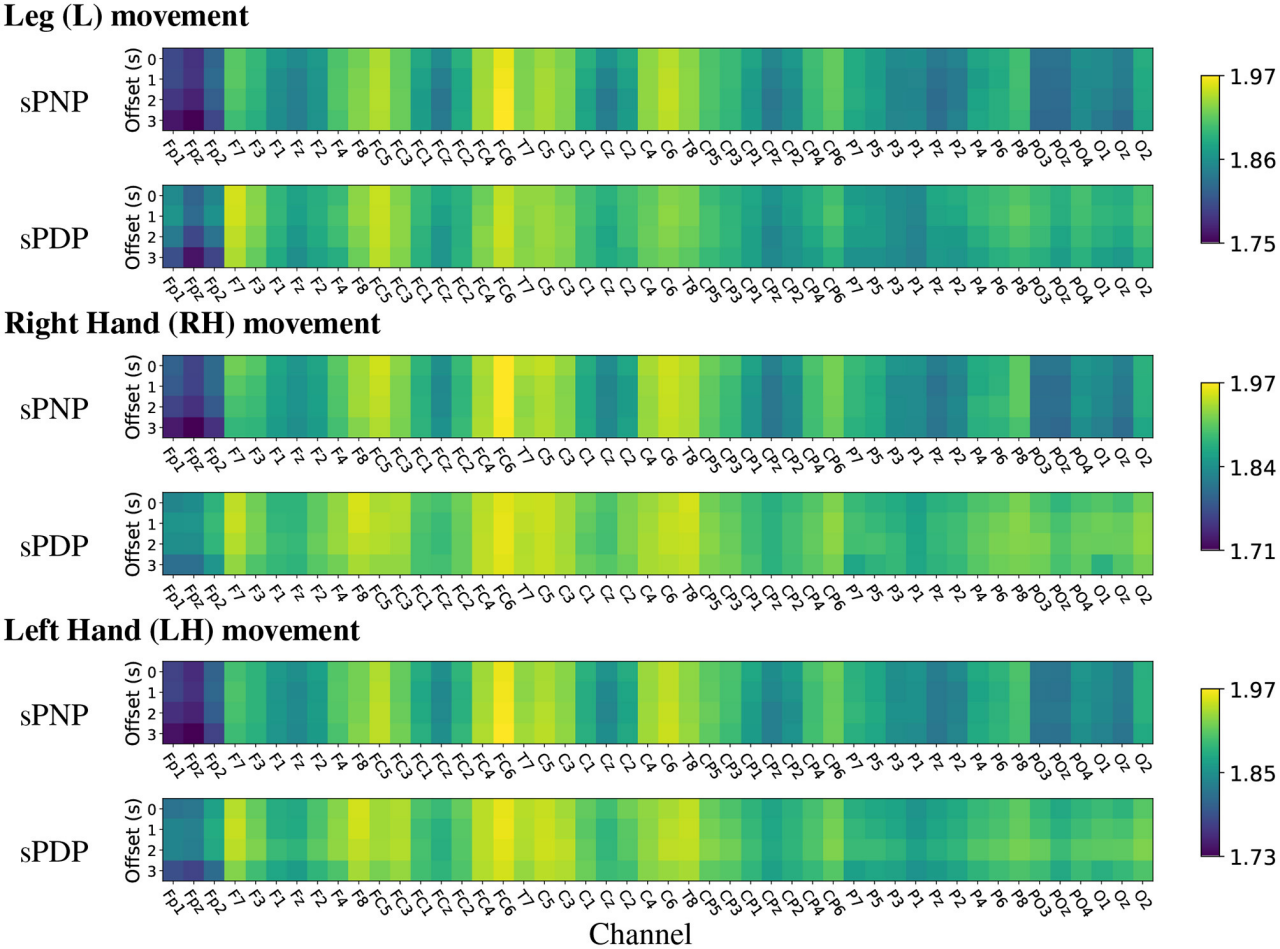
(Dataset 2) Heat maps of group-wise mean Higuchi Fractal Dimension features extracted from EEG signals recorded during movement imagination from participants with subacute SCI that did not developed pain (sPNP) and participants with subacute SCI that later developed pain (sPDP). Colours represent low (blue), medium (green), and high (yellow) mean HFD ordered by channel along the x-axis and by time offset along the y-axis.

The analysis of mean HFD on Dataset 2 indicates high complexity during MI of all limbs across the frontal-parietal lobe and in many central electrodes (see Figure 8). In both groups of participants with subacute SCI, highest mean HFD were observed at the centro-frontal electrodes and in particular over the right hemisphere. Interestingly, the lowest mean HFD were observed at the pre-frontal electrodes Fp1, Fpz, and Fp2. Often the frontal area is associated with the affective aspects of pain (Jensen et al., 2013) and our results indicate that the lowest signal complexity can be observed in the 3 most frontal electrodes for all movements in people with subacute SCI who will not develop CNP within 6 months post recording.

### 3.2. Differences in HFD Across Participant Groups

**Figure 9:**
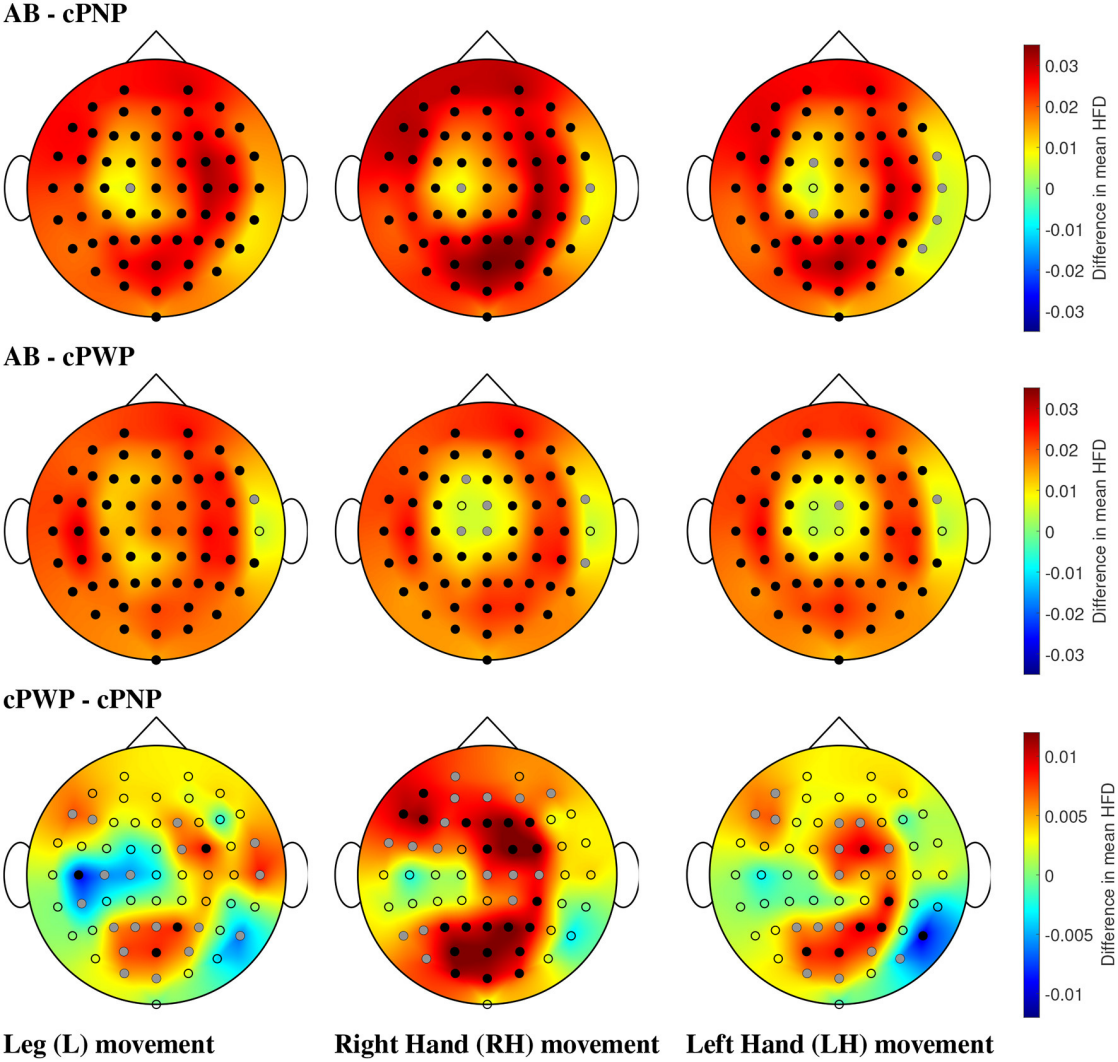
(Dataset 1) Comparison of mean HFD between able-bodied volunteers vs. participants with chronic SCI and no pain (AB - cPNP, **top**), AB vs. participants with chronic SCI and with CNP (AB - cPWP, **middle**), and cPWP vs. cPNP **(bottom)** during MI of L, RH, and LH, in the left, center, and right column, respectively, shown as an example for the time window with 2 s offset. Dots represent the locations of all 61 channels; black dots represent electrodes with statistically significant differences in mean HFD and correction for multiple comparisons, and grey dots represent electrodes with statistically significant differences (*p* = 0.05) without correction for multiple comparisons. Spatial distributions demonstrate the difference between mean HFD across each pair of groups, where red represents a large positive difference and blue indicates a large negative difference.

Analysing Dataset 1, significant differences in mean HFD were found between all pairs of groups in at least some electrodes. Visual representations of all pair-wise comparisons of mean HFD between participant groups in Figure 9 show the electrode locations at which statistically significant differences in mean HFD were found in relation to the topographic spatial distributions of differences in mean HFD at each location. Black electrodes indicate statistically significant differences after correction for multiple comparisons. Grey electrodes indicate significance before correction. All plots are representations of differences in mean HFD extracted from time windows with 2 s time offset.

AB exhibited the greatest HFD values, illustrated by large positive differences (red) in comparisons with cPNP and cPWP, respectively. Differences were found to be statistically significant (using bootstrap, *p* = 0.05, before and after Bonferroni Correction) at most electrode locations covering the whole cortex (see Figure 9, top and middle rows). Both the cPNP and cPWP groups had reduced mean HFD compared to AB across all 4 time windows (not shown). Comparing cPWP and cPNP (Figure 9, bottom row), we observed large positive differences in mean HFD, indicating higher mean HFD in the cPWP group and thus, an overall increase in the complexity of EEG signals during MI in participants with chronic SCI and chronic CNP compared to participants with chronic SCI and no CNP.

Across all limb movement imaginations (L, RH, and LH), fewest areas of statistically significant differences were seen in the comparison cPWP - cPNP. For MI of non-painful limbs (RH and LH), and in particular for the MI of the dominant right hand, the areas of largest difference between cPWP and cPNP were located over the parietal (secondary sensory) and pre-frontal cortex which are considered part of the “pain matrix” (Jensen et al., 2011). On the contrary, for painful legs, larger differences were found over the left (dominant) central cortex at C3 and C5, indicating larger EEG signal complexity over the primary motor cortex.

**Figure 10:**
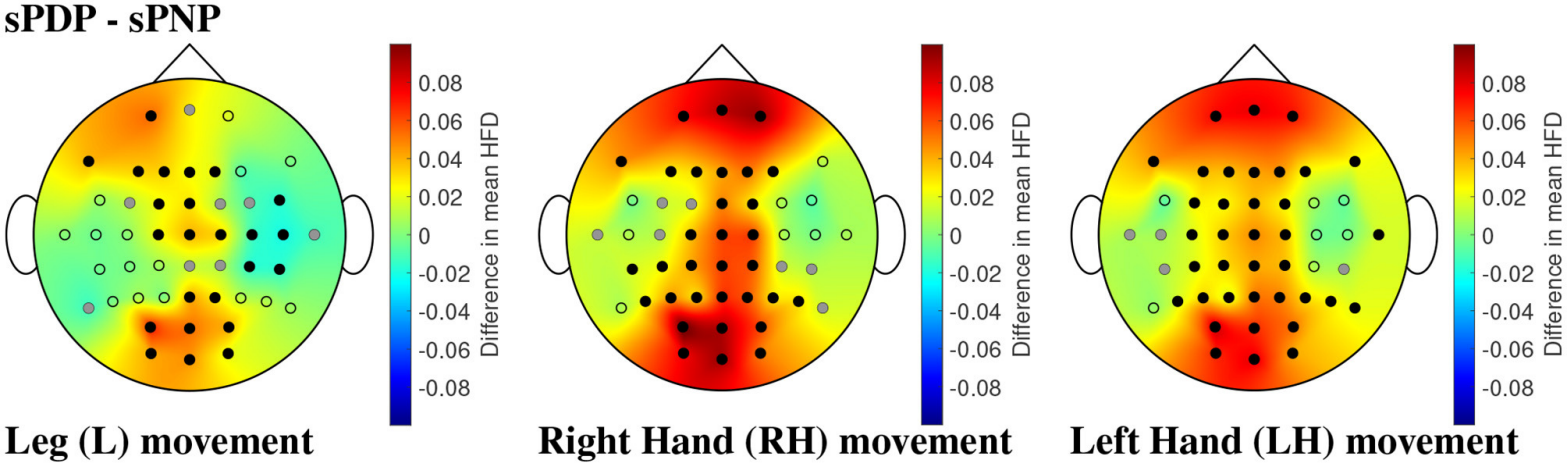
(Dataset 2) Comparisons of mean HFD between participants with subacute SCI that later developed pain (sPDP) and subacute SCI that did not developed pain (sPNP) during MI of L, RH, and LH, in the left, center, and right column, respectively, shown as an example for the time window with 2 s offset. Dots represent the locations of all 48 channels; black dots represent electrodes with statistically significant differences in mean HFD and correction for multiple comparisons, and grey dots represent electrodes with statistically significant differences (*p* = 0.05) without correction for multiple comparisons. Spatial distributions demonstrate the difference in mean HFD across groups, where red represents a large positive difference and blue indicates a large negative difference.

The same analyses were performed on Dataset 2, which showed that participants with subacute SCI who later developed pain (sPDP) have generally higher mean HFD than those who later did not develop pain (sPNP) in Figure 10. The areas of largest difference between sPDP and sPNP were located over occipital and frontal-parietal electrodes. The topographic representation of these electrodes in Figure 10 shows several locations of significant differences in mean HFD for all limb movements. During MI of legs, fewer locations with significant differences were observed than during MI of hands. Large differences in HFD values were observed in the sensorimotor cortex during MI of all limbs. Additionally, for MI of legs, there was a cluster of electrodes over the right central cortex revealing negative differences in mean HFD, indicating that HFD values were greater in sPNP than sPDP during MI of legs. These differences were also statistically significant.

### 3.3. Discrimination of Participant Groups

Can HFD features be used as diagnostic markers? Table 1 shows classification performance obtained with transferable classifiers trained on Dataset 1 to discriminate between cPWP and cPNP from examples of each type of movement imagination, respectively. It also states the hyper-parameter values optimised via nested cross-validation, i.e., the time offset, the subset of EEG channels, and the SVM-ν parameter. We observed a mean accuracy across participants ≥ 82%, which is considerably above chance level (50%, when class labels are chosen uniformly at random). Highest mean accuracy across participants was observed with leg and left hand movements, respectively. We note a large variability in accuracy across participants, indicating that classification errors are correlated across trials recorded from the same participant. At the same time, aggregating predictions across repeated trials shows a clear benefit, as can be observed from the fraction of participants for which the majority of trials were classified correctly (Table 1, column *Participants correct*), which is consistently higher than the corresponding mean accuracy. Using the classifier trained on leg movement data (Table 1, bottom row), for example, 89% participants were classified correctly after aggregating predictions across all of their trials compared to 84% mean accuracy per recording.

**Table 1:** (Dataset 1) Classification performance on cPWP vs. cPNP, evaluated separately on Right Hand (RH), Left Hand (LH), and Leg (L) data.

	**Time**	**Selected**	**Top**	**Accuracy**			**Participants**
**Limb**	**offset (s)**	**channels**	**SVM-ν**	**mean ± st. dev**.	**Sensitivity**	**Specificity**	**correct (%)**
RH	2	F2	0.15	0.82 ± 0.29	0.87	0.78	84
LH	0	F3	0.1	0.84 ± 0.28	0.83	0.84	89
L	3	F2	0.5	0.84 ± 0.29	0.95	0.71	89

*Time Offset, Selected Channels, and Top SVM-ν specify the hyper-parameters with maximum LOOCV mean accuracy. The final column Participants correct shows participant-level class prediction accuracy based on the class that was most frequently predicted across individual recordings*.

**Figure 11:**
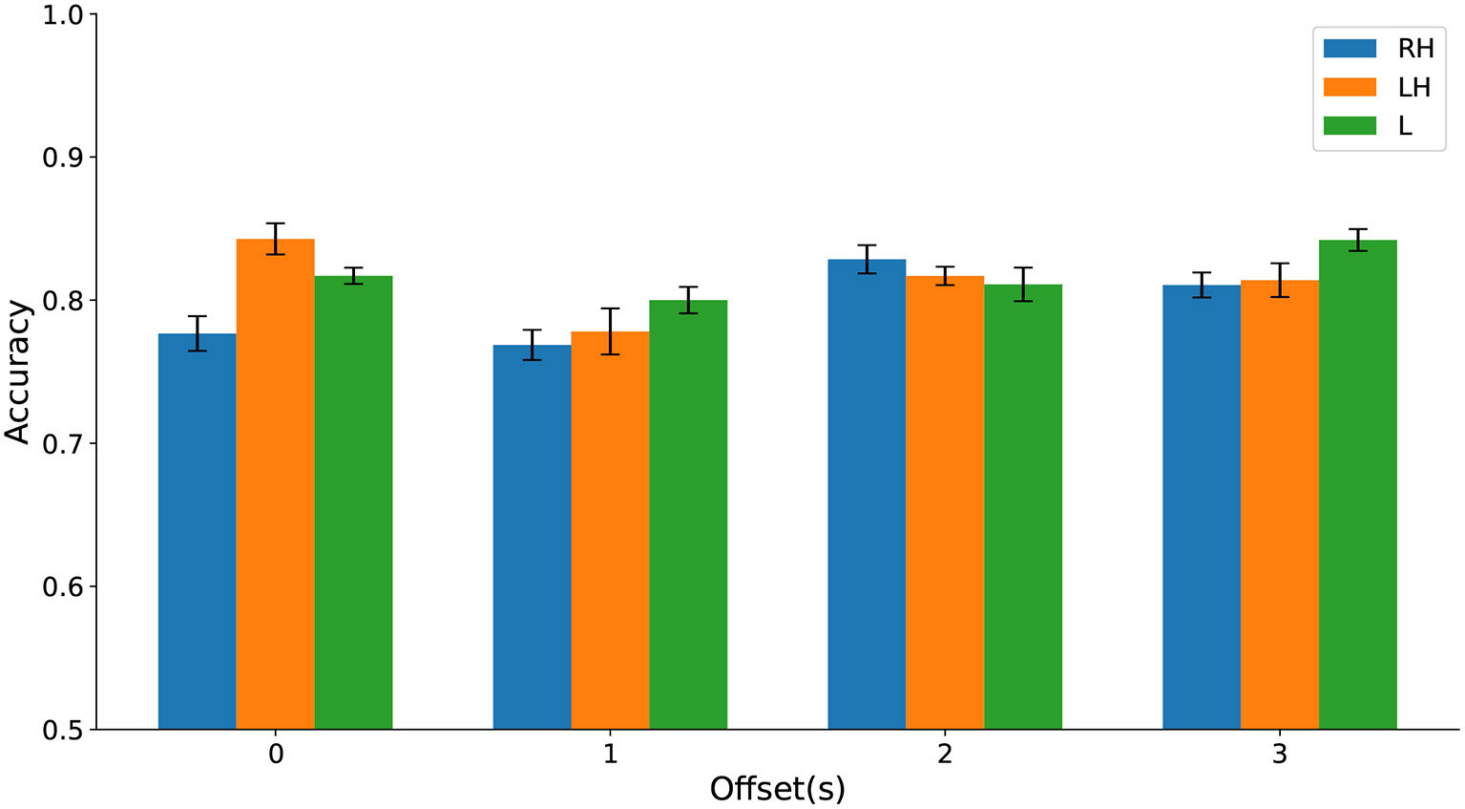
(Dataset 1) Classification accuracy evaluated on cPWP vs. cPNP separately on Right Hand (RH), Left Hand (LH), and Leg (L) data (represented by bars in different colours) varies with temporal window offset (groups along the x-axis).

Using a multiway analysis of variance (ANOVA), we found no statistically significant main effect of the time window offset or the type of movement imagination on classification accuracy and no significant interaction effect. Classification accuracies are contrasted in Figure 11 and test statistics are detailed in the Supplementary Material.

Jointly, these results strongly suggest that HFD features are useful as diagnostic markers for CNP with robust per-recording accuracy across types of movement imaginations and temporal offset from visual cue, with further improvement gained from repeated measurements. Highly discriminative features were found in the frontal area, based on data from a single electrode.

Can HFD features be used as prognostic markers for CNP in participants with subacute SCI? Table 2 shows classification performance obtained with transferable classifiers trained on Dataset 2 to discriminate between sPDP and sPNP. We observed a mean accuracy across participants of ≥ 85%. Highest mean accuracy of 88% was observed with classifiers trained on right hand (RH) and LH, respectively. The variability of classification accuracy across participants was lower than we observed on Dataset 1, cPWP vs. cPNP, and aggregating predictions across all trials from each participant resulted in all participants classified correctly using RH and L data, respectively (Table 2, column *Participants correct*).

**Table 2:** (Dataset 2) Classification performance on sPDP vs. sPNP, evaluated separately on Right Hand (RH), Left Hand (LH), and Leg (L) data.

	**Time**	**Selected**	**Top**	**Accuracy**			**Participants**
**Limb**	**offset (s)**	**channels**	**SVM-ν**	**mean ± st. dev**.	**Sensitivity**	**Specificity**	**correct (%)**
RH	2	FC6, Oz, CPz	0.3	0.88 ± 0.11	0.9	0.83	100
LH	1	FCz	0.325	0.88 ± 0.17	0.91	0.85	85
L	3	CP3	0.725	0.85 ± 0.17	0.88	0.84	100

*Time Offset, Selected Channels, and Top SVM-ν specify the hyper-parameters with maximum LOOCV mean accuracy. The final column Participants correct shows participant-level class prediction accuracy based on the class that was most frequently predicted across individual recordings*.

**Figure 12:**
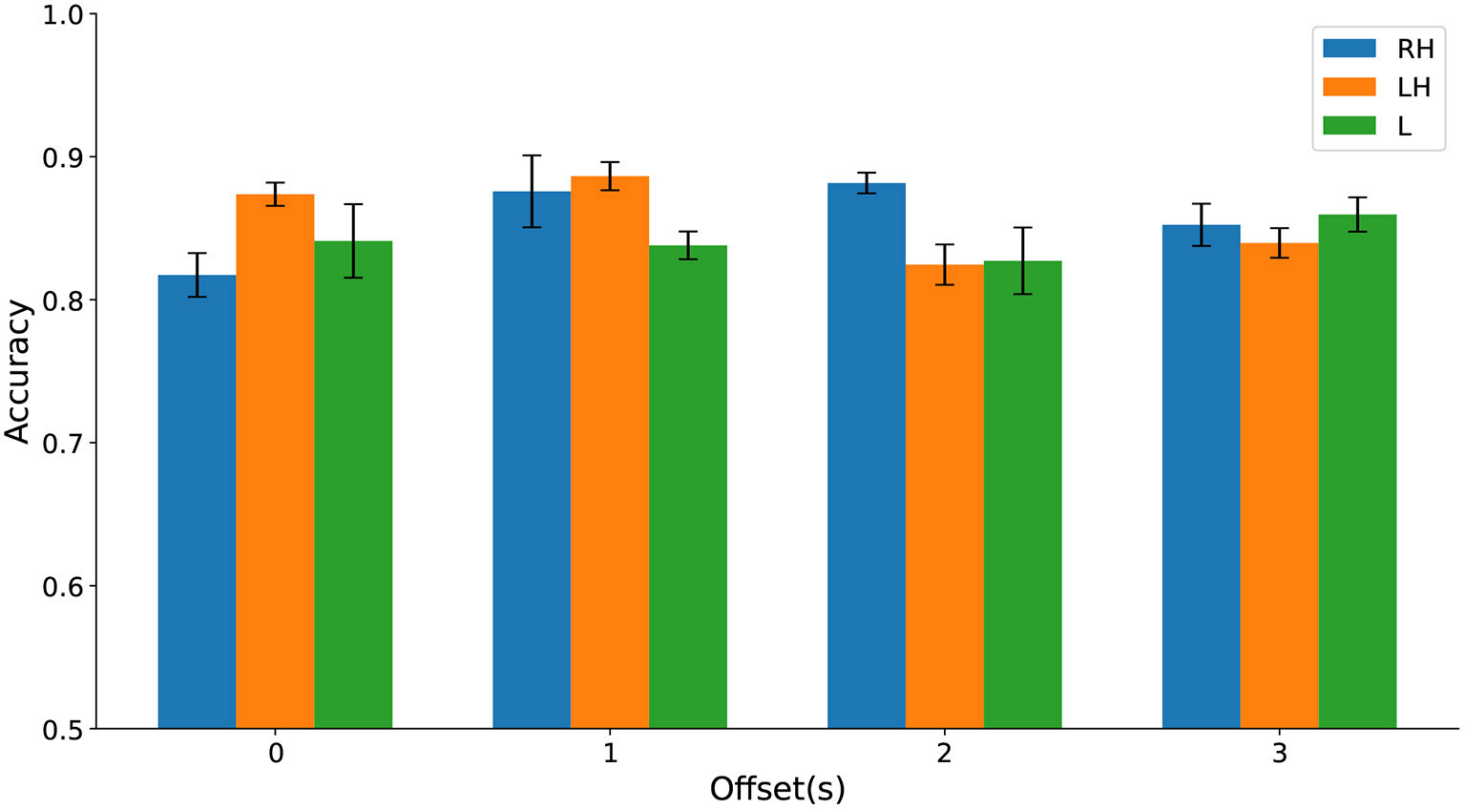
(Dataset 2) Classification accuracy evaluated on sPDP vs. sPNP separately on Right Hand (RH), Left Hand (LH), and Leg (L) data (represented by bars with different colours) varies with temporal window offset (groups along the x-axis).

Differences in classification accuracy across time windows and types of movement imagination were not significant (see Figure 12 and Supplementary Material). These results further suggest that HFD can be a useful prognostic marker for CNP. Again, up to three channels sufficed to predict pain. In comparison to discrimination between participants with chronic SCI with and without chronic CNP on Dataset 1, cPWP vs. cPNP, locations of channels were less consistent across types of movement imaginations, but equally included frontal electrodes for left and right hand movements.

The results from our exploration of whether parameters optimised for diagnostic classification (Dataset 1, cPWP vs. cPNP) can be transferred to prognostic classification (Dataset 2, sPDP vs. sPNP) are shown in Table 3. In this experiment, we observed mean accuracy of 82, 70, and 72% with RH, LH, and Leg (L) movement imaginations, respectively, which are all considerably above chance level (50%, as above). Highest accuracy was observed for RH, which originally had the most discriminating features at a frontal electrode.

**Table 3:** (Dataset 2) Classification performance on sPDP vs. sPNP using best-performing channels from (Dataset 1, cPWP vs. cPNP), evaluated separately on Right Hand (RH), Left Hand (LH), and Leg (L) data.

	**Time**	**Transferred**	**Top**	**Accuracy**			**Participants**
**Limb**	**offset (s)**	**channels**	**SVM-ν**	**mean ± st. dev**.	**Sensitivity**	**Specificity**	**correct (%)**
RH	2	F2	0.475	0.82 ± 0.36	0.97	0.59	83
LH	0	F3	0.45	0.70 ± 0.38	0.75	0.64	71
L	3	F2	0.275	0.72 ± 0.4	0.60	0.80	75

*Time Offset and Top SVM-ν specify the hyper-parameters with maximum LOOCV mean accuracy, whereas Transferred Channels specify the combination of channels identified by training on Dataset 1, discriminating between participants with chronic SCI and with chronic CNP and participants with chronic SCI and without CNP (cPWP vs. cPNP).The final column Participants correct shows participant-level class prediction accuracy based on the class that was most frequently predicted across individual recordings*.

## 4. Discussion

This study demonstrates that cortical changes due to SCI that lead to the development of CNP can be identified and classified using fractal analysis. The most significant finding is that future and chronic CNP share common features and as a result, the same classifier can be used for both. This sheds new light on pain chronification by showing that frontal areas, involved in the affective aspects of pain and believed to be influenced by long-standing pain, are affected in a much earlier phase of pain development. Another important finding is that both predictive and diagnostic markers of pain can be identified with as little as one frontal electrode, indicating a potential for more practical clinical applications.

We did not find significant differences in classification between different time windows, suggesting that HFD are not affected by the phase of movement. It would be interesting to test in the future whether discriminable HFD features could be defined in a resting state EEG because we know that non-evoked EEG contains classifiable linear features derived from frequency-band analysis (Vuckovic et al., 2018a). Smits et al. (2016) have found that, during rest, EEG-derived HFD values were sensitive to neuronal changes related to aging in healthy people and Alzheimer's disease. Additionally, resting-state EEG activity has been used to extract specific features of other neurological disorders, using non-linear techniques such as entropy Cao et al. (2018). From our results and those of other studies, it would be rational to hypothesise that resting-state EEG may contain discriminable HFD features of CNP.

Previous studies focused on neuropathic pain were based on features derived from the resting state EEG, and included greater numbers of electrodes for classification (Sarnthein et al., 2006; Vanneste et al., 2018; Vuckovic et al., 2018a) compared to the small numbers found in this study. Typically these studies use conventional oscillatory features derived from time frequency analysis for classification and have reached accuracies greater than 85%. Vanneste et al. (2018) also utilised SVMs for analysing resting-state EEG patters in people with a variety of neurological conditions such as Parkinson's disease, neuropathic pain, tinnitus and depression. They identified specific and classifiable mechanisms underlying these disorders and with respect to pain, their observations saw SVM learning to differentiate between pain and healthy control volunteers with an accuracy of 92.5%.

While, to the best of our knowledge, there has been no study defining EEG markers of neuropathic pain based on non-oscillatory features, Cao et al. (2018) developed classifiers based on entropy to identify the preictal phase of migraine, which precedes the onset of pain, and the interictal phase. They also found the most discriminable features of pain at frontal electrodes during resting-state EEG rather MI. The orbitofrontal cortex is involved in the affective aspects of chronic pain in general, which may explain why similar locations were found in the current study and Cao et al. (2018). Their study, however, only investigated frontal and occipital electrodes following advice from previous reporting that migraine patients suffer from lobe disfunction in these areas. They also utilised a relatively small selection of 4 electrodes when compared to similar studies.

Another study, also using oscillatory EEG features, but investigating specifically people with SCI, used a 32 electrode EEG montage and applied discrimination analysis and cross-correlation to allocate SCI patients with above-level and below-level NP according to their EEG peak frequency. 84.2% of all SCI participants were allocated to the correct group (Wydenkeller et al., 2009).

Tripanpitak et al. (2020) also hypothesised that features based on non-linear analysis, specifically HFD, could catch useful information about pain, more specifically evoked pain from electrical stimulation. This study also employed artificial neural networks, a classification technique, in order to predict the evoked-pain perception levels. Features from HFD were able to classify pain perception levels with above-chance level accuracies, however it was reported that combining different FD-based features, resulted in higher success rates.

We have shown that only up to 3 electrodes can suffice to predict pain with accuracy great than 80%. A frontal location is appealing, in particular, for practical applications. This is because it is typically not covered by hair enabling fast set-up with the potential for using commercially available EEG devices, most of which record EEG with few electrodes from the frontal cortex. Our study that previously applied classifiers to predict SCI CNP (Vuckovic et al., 2018a) identified the optimal numbers of EEG channels to be between 9 and 18. This was dependent on which classifier was being used and the groups being compared. With future clinical applications in mind, this study also trained the classifiers using features from the 10 best channels which were identified by examining classification errors. By doing so, accuracies were reduced but remained above chance level at between 83 and 86%. Again, these classifiers utilised oscillatory EEG behaviour.

With respect to study limitations, the first which we consider is that, although this study utilised data with comparable sample numbers to similar relevant studies, in order to generalise these pain classification results it would be necessary to increase sample numbers. It would also be beneficial to test the validity of the classifiers on unlabelled data and follow up with patients as to the status of their pain development. Secondly, we did not have access to information relating to whether any of the participants in the subacute population have developed pain since the reported study was conducted. Consequently, we do not know if some participants have been incorrectly labelled during the classification process i.e., sPNP that in fact did develop pain after study had finished reporting. Future studies should consider ensuring that pain status could be followed up. However, it is known that, in most people, CNP develops within the first 6 months post-injury (Siddall et al., 2003). As a result, we believe that by following up patients for 6 months we managed to identify correctly most participants who later developed pain. Additionally, the heterogeneity of the data was not addressed in this study. Vuckovic et al. (2018b) compared descriptive (pain) and demographic factors (age, level of injury, time after injury) between the groups using analysis of variance (ANOVA) and compared completeness of injury using non-parametric testing. There were no significant between-group differences in age, time post-injury or ASIA level. The patient pain scores, and level of injuries differed significantly between the groups. Also, the sPNP group had injuries 8 levels lower than the sPDP group. Age and completeness of injury were matched across groups in the subacute dataset but the level of injury was not matched between sPNP and sPDP groups. Previous analysis on this data also indicated that it was not possible to extract features to measure the influence that level of injury had on the presence or development of pain although some believe there is no correlation between level or completeness of injury and pain (Siddall et al., 2003). Finnerup et al. (2014) have also reported on the effects of demographic factors and injury characteristics on the development of neuropathic pain with greater sample sizes than that in the current study. They interestingly observed that injury characteristics, such as completeness and neurological level, could not predict at- or below-level neuropathic pain. Comparisons of this nature, between groups in Dataset 1 (AB, cPWP, cPNP) have not been performed. Given the small sample sizes of both datasets, exploring the heterogeneity within these groups would require further data but could provide potentially valuable clinical information as to those more likely to develop pain. We plan to address this in future research.

In conclusion, this study provides supporting evidence for the potential of non-oscillatory non-linear features of EEG as diagnostic and prognostic biomarkers for neurological disorders and it suggests a new perspective on pain chronification showing that frontal areas, involved in the affective aspects of pain, are affected in a much earlier phase of pain development than is commonly believed. In future work we will seek to confirm our findings on a larger dataset involving more subacute participants with SCI and explore pain chronification by training and analysing machine learning models to predict the delay after which participants with asymptomatic CNP start showing symptoms.

## Data Availability Statement

The raw data supporting the conclusions of this article will be made available by the authors, without undue reservation.

## Ethics Statement

This study was reviewed and approved by the National Health Service ethical committee for the patient groups and from the university ethical committee for the able-bodied group. Written informed consent was obtained from all participants.

## Author Contributions

CC, SS, and AV conceived of the presented idea. CC and SS designed the model and the computational framework. MF and MP coordinated data collection. CC and KA implemented the analysis and performed the computations under supervision of SS and AV. All authors discussed the results and contributed to the final manuscript.

## Conflict of Interest

The authors declare that the research was conducted in the absence of any commercial or financial relationships that could be construed as a potential conflict of interest.

## Publisher's Note

All claims expressed in this article are solely those of the authors and do not necessarily represent those of their affiliated organizations, or those of the publisher, the editors and the reviewers. Any product that may be evaluated in this article, or claim that may be made by its manufacturer, is not guaranteed or endorsed by the publisher.

## Footnotes

^1^HFD implementation: https://github.com/inuritdino/HiguchiFractalDimension.

^2^Sklearn ν-SVC implementation: https://scikit-learn.org/stable/modules/generated/sklearn.svm.NuSVC.html.
