# Supplementary material for: Markers of Central Neuropathic Pain in Higuchi Fractal Analysis of EEG Signals From People With Spinal Cord Injury
Source: Front Neurosci. 2021 Aug 26;15:705652. doi: 10.3389/fnins.2021.705652 (PMC8427815; doi:10.3389/fnins.2021.705652)
Supplement: Supplementary file 1 [file Data_Sheet_1.PDF]

Markers of Central Neuropathic Pain in Higuchi Fractal Analysis of EEG Signals from People with Spinal Cord Injury

SUPPLEMENTARY MATERIAL

This Section provides analysis details of the statistical significance tests on *diagnostic* and *prognostic* participant group classification results discussed in Section 3.3 of this paper. 2-way analysis of variance (ANOVA) was performed on the distributions of mean per-participant classification accuracy with time window offset and type of movement imagination as independent variables.

Table S1. (Dataset 1) 2-way ANOVA test statistics observed with *diagnostic* classification results on Dataset 1, cPWP vs. cPNP, where classification accuracy is the dependent variable, and time window offset (offset) and type of movement imagination (limb) are independent variables.

Source	*Sum Sq.*	*d.f.*	*Mean Sq.*	*F*	*p*
offset	0.052	3	0.01732	0.22	0.8849
limb	0.0224	2	0.01119	0.14	0.8696
offset*limb	0.033	6	0.0055	0.07	0.9987

Table S1 shows the test statistics observed with diagnostic classification results on Dataset 1, cPWP vs. cPNP. The null hypothesis that the independent variables or their interaction have no effect on classification accuracy could not be rejected ($p > 0.05$).

Table S2. (Dataset 2) 2-way ANOVA test statistics observed with *prognostic* classification results on Dataset 2, sPDP vs. sPNP, where classification accuracy is the dependent variable, and time window offset (offset) and type of movement imagination (limb) are independent variables.

Source	*Sum Sq.*	*d.f.*	*Mean Sq.*	*F*	*p*
offset	0.01692	3	0.00564	0.09	0.9663
limb	0.00672	2	0.00336	0.05	0.9487
offset*limb	0.06289	6	0.01048	0.16	0.9858

Table S2 shows the test statistics observed with prognostic classification results on Dataset 2, sPDP vs. sPNP. The null hypothesis that the independent variables or their interaction have no effect on classification accuracy could not be rejected ($p > 0.05$).
